# Prediabetes and the risk of incident chronic kidney disease in adults: A systematic review and meta-analysis

**DOI:** 10.17305/bb.2026.13524

**Published:** 2026-01-23

**Authors:** Sitian Fang, Jinjing Huang, Yanxia Chen

**Affiliations:** 1 Huankui Academy, Nanchang University, Nanchang, China; 2 Department of Nephrology, Second Affiliated Hospital, Jiangxi Medical College, Nanchang University, Nanchang, China

**Keywords:** Prediabetes, chronic kidney disease, risk factor, incidence, meta-analysis

## Abstract

The relationship between prediabetes and chronic kidney disease (CKD) remains ambiguous, with varying results across cohort studies. This meta-analysis aimed to assess whether prediabetes is linked to an increased risk of developing incident CKD in the general adult population. A comprehensive search was conducted in PubMed, Embase, and Web of Science from inception to September 28, 2025, for longitudinal observational studies that evaluated CKD risk in individuals with prediabetes compared to those with normoglycemia. Prediabetes was defined by impaired fasting glucose (IFG), impaired glucose tolerance (IGT), elevated glycated hemoglobin (HbA1c), or a combination of these criteria. Pooled risk ratios (RRs) with 95% confidence intervals (CIs) were calculated using a random-effects model. Fifteen cohorts comprising 2,854,724 participants were included in the analysis. The results indicated that prediabetes was significantly associated with an increased risk of incident CKD (RR: 1.21, 95% CI: 1.12–1.31; *I*^2^ ═ 90%). Subgroup analyses revealed that the association was not significantly influenced by the definitions of prediabetes, study design, demographic characteristics of the population, follow-up duration, or study quality scores (*P* for subgroup difference all > 0.05). Meta-regression analysis suggested that a higher mean age of the population was inversely correlated with the observed effect size for the relationship between prediabetes and CKD risk (coefficient ═ −0.030, *P* ═ 0.004; adjusted R^2^ ═ 67%). In conclusion, prediabetes is associated with a modestly elevated risk of developing CKD in the general population, with a potentially stronger correlation observed in younger individuals. These findings indicate an association rather than causality and suggest that early glycemic dysregulation may be linked to subsequent renal risk prior to the onset of overt diabetes.

## Introduction

Chronic kidney disease (CKD) represents a significant global health challenge, impacting approximately 10% of the adult population and resulting in considerable morbidity, mortality, and healthcare costs [1, 2]. The progression of CKD to end-stage renal disease often necessitates dialysis or kidney transplantation and heightens the risk of cardiovascular complications [3]. Despite advancements in treatment modalities, including renin–angiotensin–aldosterone system inhibition and the management of glycemia and blood pressure, the long-term prognosis for CKD remains unfavorable [4]. Therefore, the early identification and mitigation of modifiable risk factors are essential to slow disease progression and reduce associated complications. Among these risk factors, hyperglycemia is a well-established contributor to diabetic kidney disease, accounting for approximately one-third of CKD cases globally [5]. However, the impact of milder dysglycemia, below the diabetic threshold, on early kidney injury is not fully understood.

Prediabetes, an intermediate metabolic state between normoglycemia and diabetes mellitus, is increasingly recognized as a high-risk condition for the development of diabetes and cardiovascular disease [6, 7]. It is typically characterized by impaired fasting glucose (IFG), impaired glucose tolerance (IGT), or elevated glycated hemoglobin (HbA1c) levels, indicating subtle disruptions in insulin secretion and resistance [6]. Emerging evidence indicates that prediabetes may already have detrimental effects on renal microvasculature, potentially through mechanisms such as low-grade inflammation, endothelial dysfunction, oxidative stress, and glomerular hyperfiltration [8, 9]. These processes may initiate subclinical kidney injury even prior to the onset of overt diabetes, thereby linking metabolic dysregulation with CKD [8, 9]. A previous meta-analysis conducted in 2016 identified a modest yet significant association between prediabetes and an increased risk of CKD [10]. However, many of the studies included were primarily designed to assess metabolic syndrome, which may introduce confounding variables such as obesity, hypertension, and dyslipidemia [10]. Since that time, several large-scale cohort studies with enhanced diagnostic accuracy and extended follow-up have been published, necessitating an updated synthesis of the literature [11–21]. Consequently, this systematic review and meta-analysis aims to provide a comprehensive assessment of the association between prediabetes and the risk of incident CKD in the general adult population, including subgroup and meta-regression analyses to investigate potential sources of heterogeneity and population-specific effects.

## Materials and methods

The methodology of this meta-analysis adhered to the Preferred Reporting Items for Systematic Reviews and Meta-Analyses (PRISMA) 2020 guidelines [22] and the Cochrane Handbook for Systematic Reviews and Meta-Analyses [22], encompassing protocol development, data collection, statistical procedures, and reporting. The protocol was prospectively registered in International Prospective Register of Systematic Reviews (PROSPERO) (ID: CRD420251180619).

### Literature search

A comprehensive search of PubMed, Embase, and Web of Science was conducted to identify eligible studies. The search strategy employed the following term groups: (1) “prediabetes” OR “pre-diabetes” OR “prediabetic” OR “pre-diabetic” OR “prediabetic state” OR “borderline diabetes” OR “impaired fasting glucose” OR “impaired glucose tolerance” OR “IFG” OR “IGT”; (2) “chronic kidney disease” OR “CKD” OR “glomerular filtration rate” OR “renal function” OR “chronic renal failure”; (3) “cohort” OR “prospective” OR “retrospective” OR “follow” OR “followed” OR “follow-up” OR “longitudinal” OR “risk” OR “incidence.” Only full-text, peer-reviewed articles published in English and involving human subjects were considered eligible. References of relevant reviews and original reports were manually examined for additional studies. The search encompassed all records from database inception up to September 28, 2025. The detailed search strategy for each database is presented in Supplemental File 1.

### Inclusion and exclusion criteria

The selection of studies was guided by the PICOS framework:

- **Population (P):** Adults (≥ 18 years) from the general population without baseline CKD, confirmed through clinical or laboratory assessment.
- **Intervention/exposure (*I*):** Prediabetes, defined according to established diagnostic thresholds for IFG, IGT, mildly elevated HbA1c, or their combination. Due to the lack of a universally accepted hierarchy demonstrating the superiority of any single definition for predicting CKD risk, all validated prediabetes definitions were deemed eligible for the primary analysis.
- **Comparison (C):** Participants with normoglycemia served as the reference group.
- **Outcomes (O):** Incident CKD diagnosed according to the criteria of the original studies, generally defined as a decline in estimated glomerular filtration rate (eGFR) to < 60 mL/min/1.73 m^2^ and/or the presence of albuminuria, with a minimum follow-up duration of 1 year. Definitions of albuminuria were study-specific and typically corresponded to moderately increased albuminuria (A2) or higher, although precise thresholds were not consistently reported across cohorts.
- **Study design (S):** Longitudinal follow-up studies, including prospective or retrospective cohort studies, nested case–control studies, and post-hoc analyses of randomized controlled trials (RCTs) that provided baseline glycemic classification and subsequent CKD outcomes.

Exclusion criteria included reviews, meta-analyses, editorials, preclinical studies, research involving pediatric populations, cross-sectional studies, studies not involving the general population, those that did not examine prediabetes, lacked controls of normoglycemia, or those that did not report CKD incidence. Additionally, studies focused on metabolic syndrome were excluded due to their “hyperglycemia” component not consistently distinguishing prediabetes from undiagnosed diabetes, reflecting multiple metabolic factors and complicating the isolation of the independent effect of prediabetic glycemia on CKD risk. In cases of overlapping populations, the analysis incorporated the study with the largest sample size.

### Study quality evaluation and data collection

Two investigators conducted the literature search, screening, quality evaluation, and data extraction independently, resolving disagreements through consultation with the corresponding author. Study quality was assessed using the Newcastle–Ottawa Scale (NOS) [23], which evaluates cohort selection, control of confounding, and outcome ascertainment. The NOS assigns scores from 1–9, with higher scores indicating better quality; studies scoring ≥ 7 were classified as high quality. Extracted data encompassed study details (first author, year, design, country), participant information (population source, sample size, age, sex, mean body mass index [BMI] at baseline), exposure measures (diagnostic criteria for prediabetes and the number of patients with prediabetes at baseline), follow-up duration, outcome definitions (criteria for CKD diagnosis and the number of patients with newly developed CKD during follow-up), and covariates considered in the adjusted analyses of prediabetes and CKD risk.

### Statistics

We evaluated the association between prediabetes and incident CKD in the general adult population by pooling risk ratios (RRs) with corresponding 95% confidence intervals (CIs) comparing participants with prediabetes to those with normoglycemia at baseline. Effect estimates reported as hazard ratios were considered equivalent to RRs. When odds ratios (ORs) were presented, data were converted to relative risks for the meta-analysis using the formula: RR = OR / (1 -- pRef + pRef × OR), where pRef represents the prevalence of the outcome in the reference group (normoglycemia group) [24]. For each study, the most fully adjusted model was preferentially extracted to minimize confounding. If a study reported multiple definitions of prediabetes within the same population (e.g., IFG, IGT, or mildly elevated HbA1c), only one effect estimate was selected to prevent participant duplication and maintain statistical independence [22]. As no definitive evidence supports the superiority of any single prediabetes definition in predicting CKD risk, all definitions were considered clinically valid. In such instances, the RR with the largest effect size was selected to represent the maximum reported risk signal for that cohort. The robustness of this choice was further examined through prespecified subgroup analyses stratified by prediabetes definition. RRs and their standard errors were derived from reported 95% CIs or *P*-values and then log-transformed to stabilize variance and normalize the distribution [22]. Between-study heterogeneity was assessed using the Cochrane *Q* test, the *I*^2^ statistic, and the between-study variance (τ^2^). Thresholds of < 25%, 25%–75%, and > 75% for *I*^2^ were used to indicate low, moderate, and high heterogeneity, respectively [25]. Pooled effect estimates were calculated using a random-effects model, which incorporates τ^2^ to account for between-study variability [22]. To enhance interpretation in the presence of substantial heterogeneity, a 95% prediction interval (PI) was additionally calculated for the primary analysis, reflecting the expected range of true effects in future comparable populations [22]. To test robustness, sensitivity analyses were conducted by sequentially omitting individual studies [26]. Prespecified subgroup analyses further examined whether study-level characteristics influenced the findings, including the definition of prediabetes, study design (prospective vs. retrospective), mean ages of the patients, proportions of men, follow-up durations, diagnostic criteria for CKD, and study quality scores in NOS. Median values of continuous variables were used to define subgroup cutoffs. Additionally, univariate meta-regression analyses were applied to explore whether continuous variables (e.g., mean age, proportion of men, mean BMI at baseline, follow-up length, and NOS score) modified the association [22]. Subgroup analyses, in conjunction with univariate meta-regression based on study-level characteristics, were conducted in an exploratory manner to generate hypotheses regarding potential effect modifiers. Potential publication bias was assessed through visual inspection of funnel plots and Egger’s regression test [27]. A *P*-value < 0.05 was deemed statistically significant. All analyses were performed using RevMan (version 5.3, Cochrane Collaboration, Oxford, UK) and Stata (version 17.0, StataCorp, College Station, TX, USA).

## Results

### Study inclusion

**Figure 1.**
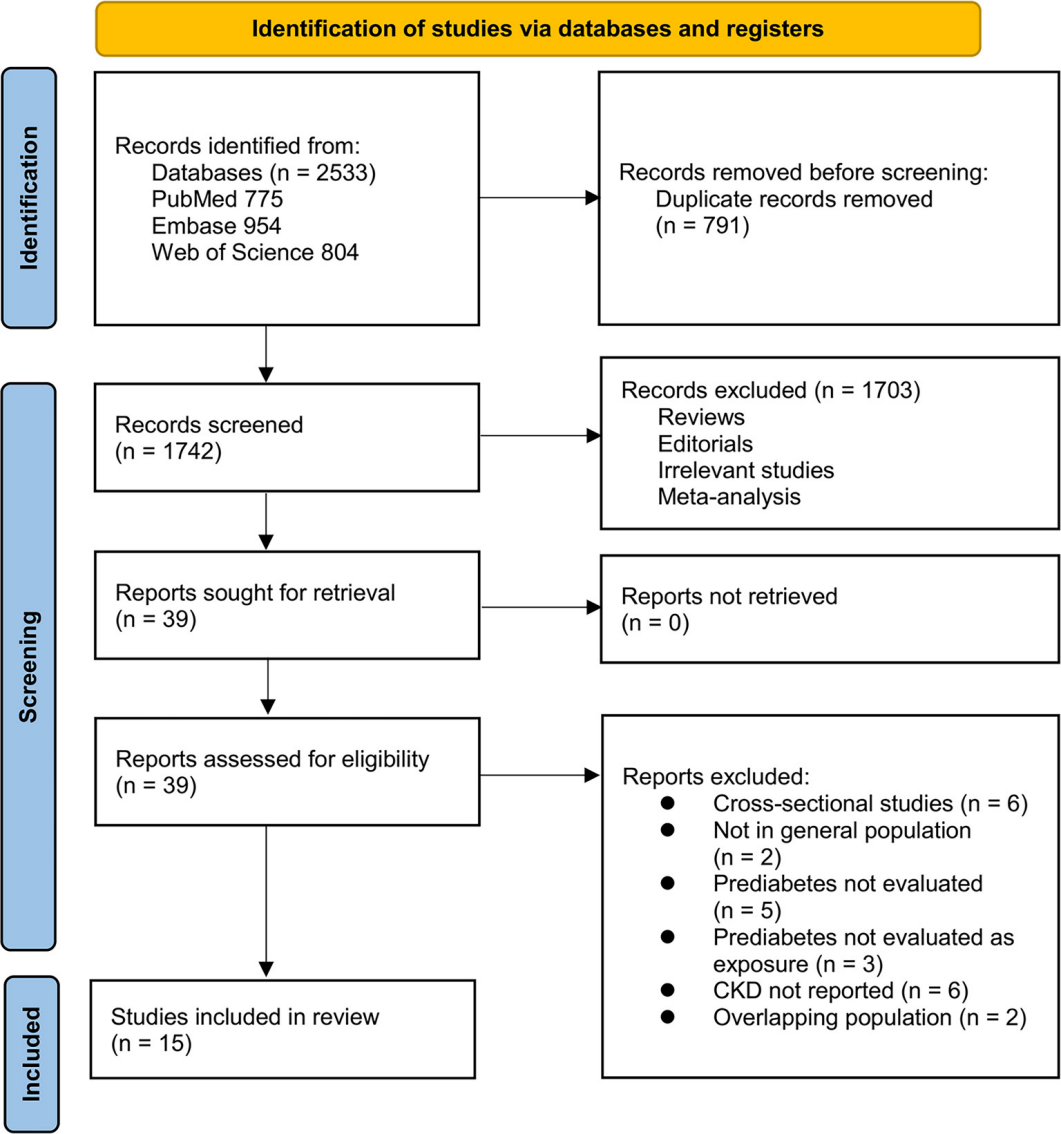
**Flow diagram illustrating the study selection process**.

The study selection process is illustrated in Figure 1. A total of 2,533 records were retrieved from three databases, with 791 duplicates eliminated. Following the screening of titles and abstracts, 1,703 articles were excluded for not meeting the eligibility criteria. The remaining 39 full-text papers were independently assessed by two reviewers, leading to the exclusion of 24 studies as detailed in Figure 1. Ultimately, 15 studies were included in the quantitative synthesis [11–21, 28–31].

### Summarized study characteristics

Table 1 summarizes the main characteristics of the included studies. A total of 15 cohort studies published between 2005 and 2025 were incorporated, comprising 9 prospective cohorts [14, 15, 17, 18, 21, 28–31] and 6 retrospective cohorts [11–13, 16, 19, 20]. These studies were conducted across various regions, including the United States, United Kingdom, Germany, Norway, Japan, China, South Korea, and Spain. The study populations were primarily derived from community-based or general adult populations without CKD at baseline. Overall, 2,854,724 adults were included in this meta-analysis. The mean age of participants ranged from 33.8–61.0 years, and the proportion of men varied between 32.9% and 100%. The mean BMI of the included subjects ranged from 22.5–28.9 kg/m^2^. Prediabetes was defined using one or more standard diagnostic criteria, including IFG, IGT, mildly elevated HbA1c, or their combinations across all studies. Accordingly, 734,770 (25.7%) of the included subjects had prediabetes at baseline. The average follow-up duration ranged from 1.7–15.0 years, during which CKD outcomes were primarily ascertained through eGFR < 60 mL/min/1.73 m^2^ in 10 studies [14, 15, 18–21, 28–31], eGFR < 60 mL/min/1.73 m^2^ and/or proteinuria in 4 studies [11–13, 16], and the International Classification of Disease codes in another study [17]. A total of 63,055 (2.2%) participants experienced new-onset CKD during follow-up. Multivariate analyses were employed in all included studies when evaluating the association between prediabetes and CKD risk, adjusting for key confounders such as age, sex, BMI, baseline eGFR, blood pressure, lipid levels, smoking status, medication use, comorbidities, and lifestyle factors to varying degrees.

### Study quality evaluation

The quality of the included studies was evaluated using the NOS, summarized in Table 2. The total NOS scores ranged from 8–9, indicating overall high methodological rigor among the included studies. Five studies [14, 18, 21, 28, 30] achieved the maximum score of 9, reflecting excellent design and follow-up. Ten studies scored 8, primarily due to poor representativeness of the exposed cohort [11, 13, 19, 20], less optimal assessment of outcomes [17], inadequate follow-up duration [12, 15, 16, 31], and slightly limited follow-up adequacy [29]. Overall, all included studies were deemed of good quality, with low risk of selection and attrition bias, supporting the reliability and validity of the pooled findings regarding the association between prediabetes and CKD risk.

### Meta-analysis

**Figure 2.**
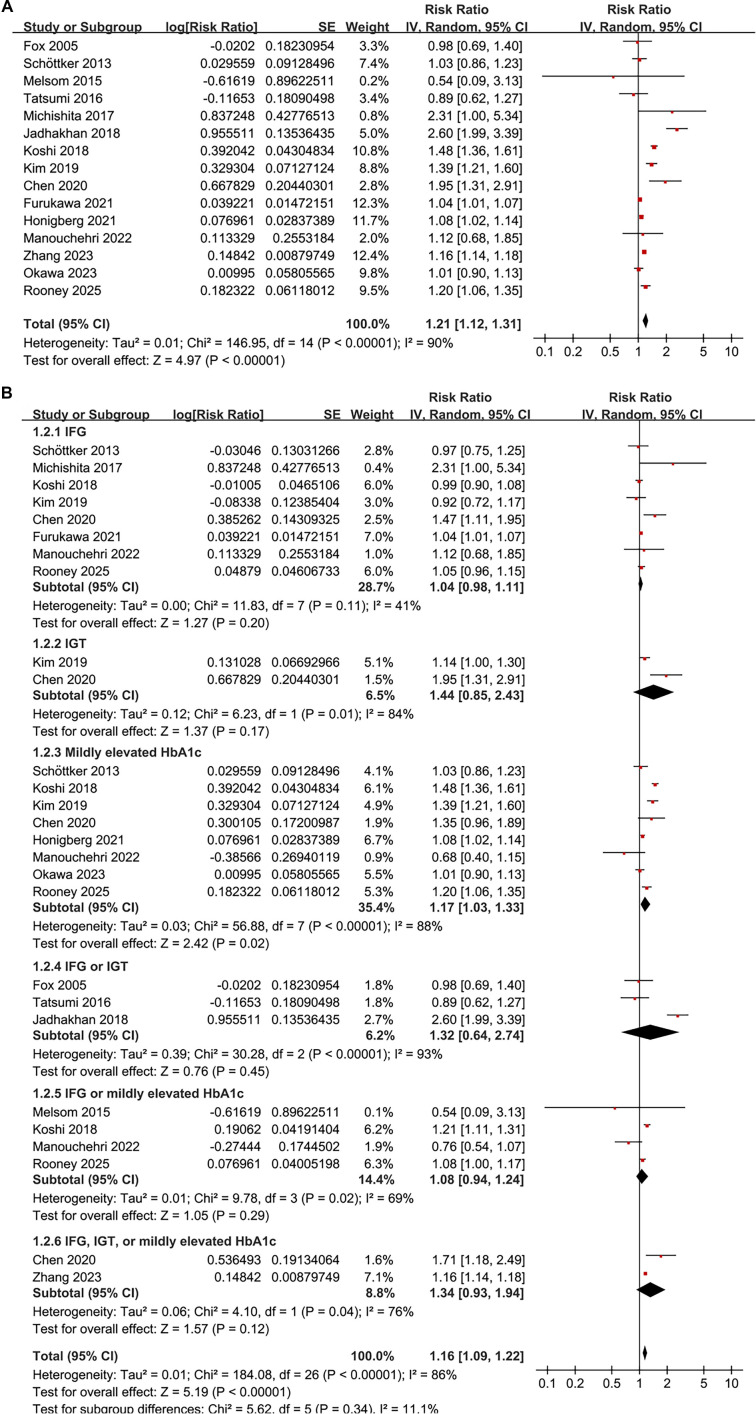
**Forest plots of the association between prediabetes and incident CKD in adults.** (A) Overall random-effects meta-analysis of 15 cohort studies comparing prediabetes versus normoglycemia, showing an increased CKD risk (pooled RR = 1.21, 95% CI 1.12–1.31; *P* < 0.001) with substantial heterogeneity (*I*^2^ ═ 90%; τ^2^ ═ 0.01) and a 95% prediction interval of 1.01–1.47. (B) Random-effects subgroup analyses stratified by prediabetes definition (IFG, IGT, mildly elevated HbA1c, and combined definitions) showing no evidence of differences between definitions (*P* for subgroup differences = 0.34); a significant association was observed only for mildly elevated HbA1c (RR = 1.17, 95% CI 1.03–1.33; *P* ═ 0.02; *I*^2^ ═ 88%). Abbreviations: RR: Risk ratio; CI: Confidence interval; CKD: Chronic kidney disease; IFG: Impaired fasting glucose; IGT: Impaired glucose tolerance; HbA1c: Glycated hemoglobin A1c.

The combined results from 15 cohorts [11–21, 28–31] demonstrate that prediabetes is associated with an increased risk of CKD in the general population compared to individuals with normoglycemia (RR: 1.21, 95% CI: 1.12–1.31; *P* < 0.001), exhibiting substantial heterogeneity between studies (*I*^2^ ═ 90%; τ^2^ ═ 0.01; Figure 2A). The corresponding 95% prediction interval ranged from 1.01–1.47, indicating considerable variability in the strength of the association across different populations. Exclusion of individual studies sequentially did not materially alter the findings, with pooled RRs ranging from 1.16–1.25 (all *P* < 0.05).

### Subgroup analysis

**Figure 3.**
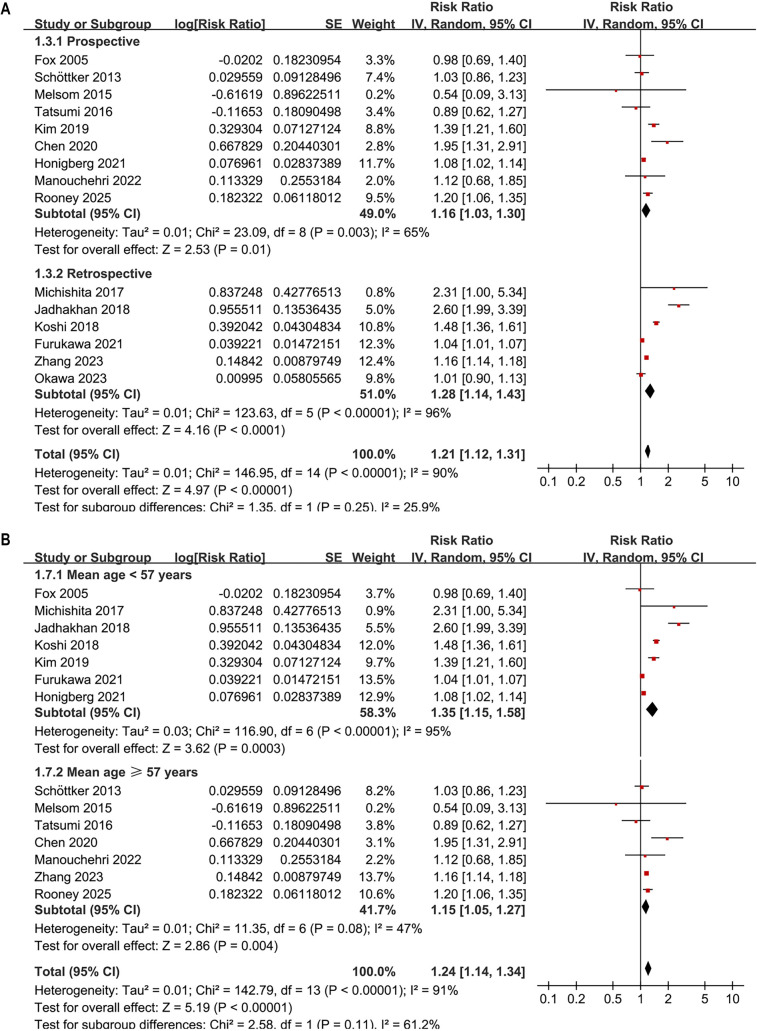
**Forest plots of subgroup analyses examining the association between prediabetes (vs. normoglycemia) and incident CKD in adults using an inverse-variance random-effects model.** (A) Stratified by study design, showing comparable pooled associations in prospective cohorts (RR = 1.16, 95% CI 1.03–1.30; *P* ═ 0.01; *I*^2^ ═ 65%) and retrospective cohorts (RR = 1.28, 95% CI 1.14–1.43; *P* < 0.0001; *I*^2^ ═ 96%), with no evidence of between-subgroup differences (*P* for subgroup differences = 0.25). (B) Stratified by mean participant age, with pooled RRs of 1.35 (95% CI 1.15–1.58; *P* ═ 0.0003; *I*^2^ ═ 95%) for studies with mean age < 57 years and 1.15 (95% CI 1.05–1.27; *P* ═ 0.004; *I*^2^ ═ 47%) for mean age ≥ 57 years (*P* for subgroup differences = 0.11). Abbreviations: RR: Risk ratio; CI: Confidence interval; CKD: Chronic kidney disease.

**Figure 4.**
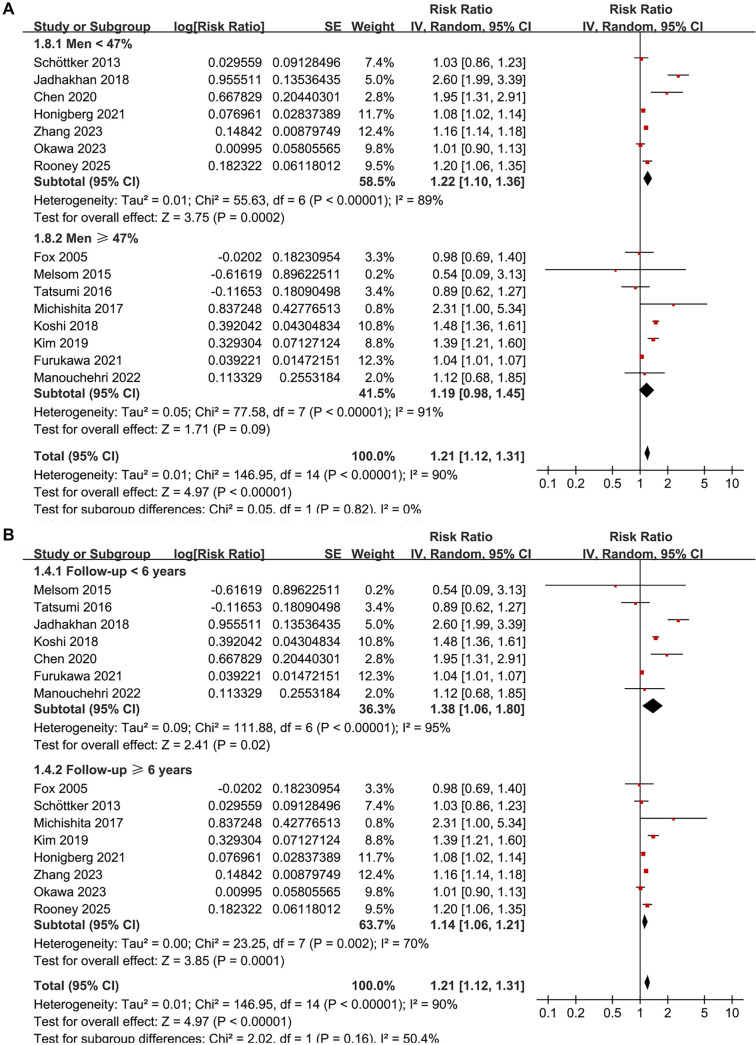
**Forest plots of subgroup analyses assessing the association between prediabetes (vs. normoglycemia) and incident CKD in adults using an inverse-variance random-effects model.** (A) Stratified by the proportion of men in the cohort (<47% vs. ≥47%), showing comparable pooled effects (RR = 1.22, 95% CI 1.10–1.36; *P* ═ 0.0002; *I*^2^ ═ 89% and RR = 1.19, 95% CI 0.98–1.45; *P* ═ 0.09; *I*^2^ ═ 91%), with no evidence of between-subgroup differences (*P* for subgroup differences = 0.82). (B) Stratified by follow-up duration (<6 vs. ≥6 years), with pooled RRs of 1.38 (95% CI 1.06–1.80; *P* ═ 0.02; *I*^2^ ═ 95%) and 1.14 (95% CI 1.06–1.21; *P* ═ 0.0001; *I*^2^ ═ 70%), respectively (*P* for subgroup differences = 0.16). Abbreviations: RR: Risk ratio; CI: Confidence interval; CKD: Chronic kidney disease.

**Figure 5.**
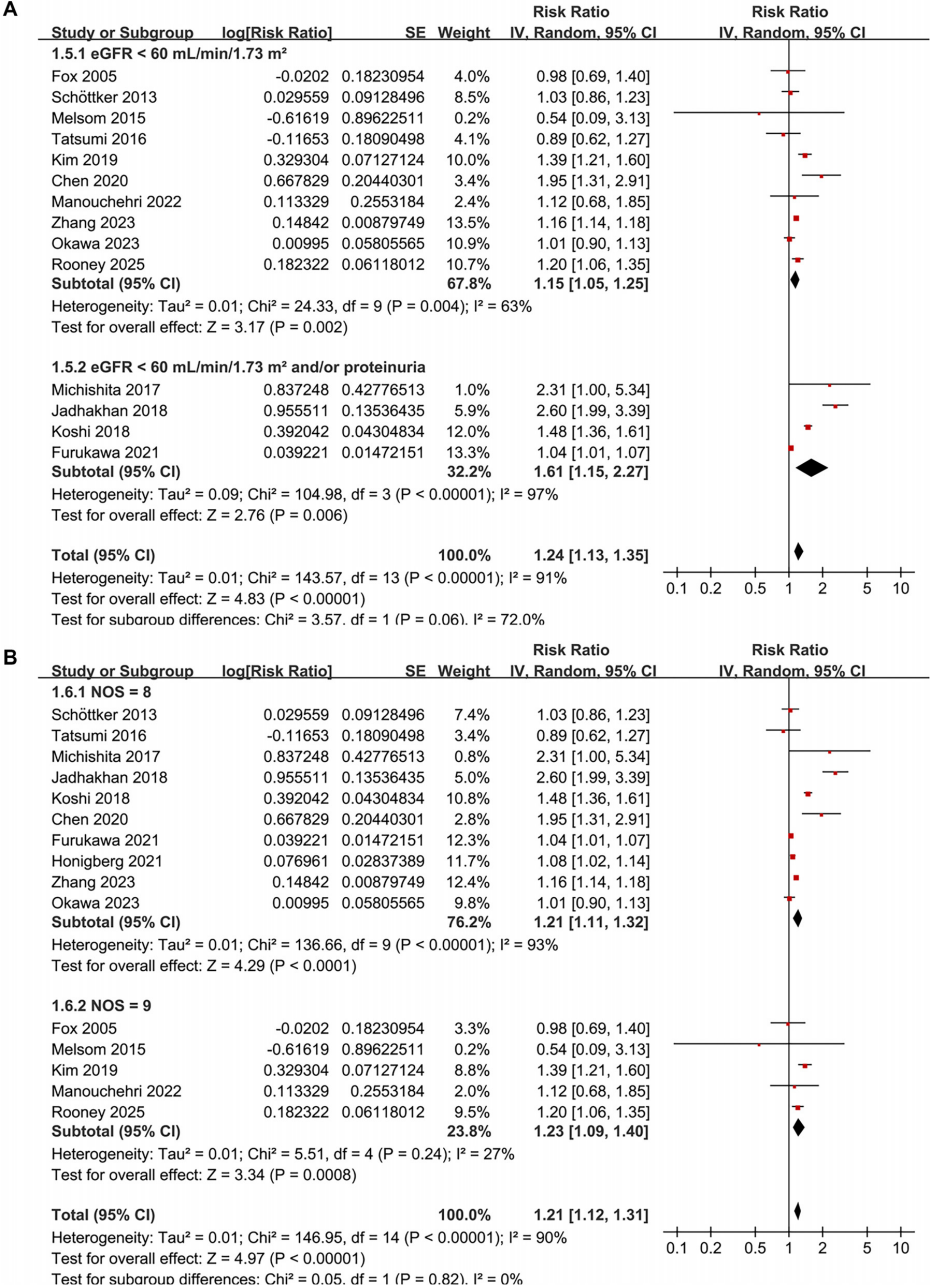
**Forest plots of subgroup analyses evaluating the association between prediabetes (vs. normoglycemia) and incident CKD in adults using an inverse-variance random-effects model.** (A) Stratified by CKD diagnostic criteria, showing a stronger pooled association in studies defining CKD as eGFR < 60 mL/min/1.73 m^2^ and/or proteinuria (RR = 1.61, 95% CI 1.15–2.27; *P* ═ 0.006; *I*^2^ ═ 97%) compared with those defining CKD as eGFR < 60 mL/min/1.73 m^2^ alone (RR = 1.15, 95% CI 1.05–1.25; *P* ═ 0.002; *I*^2^ ═ 63%), although the between-subgroup difference did not reach statistical significance (*P* for subgroup differences = 0.06). (B) Stratified by study quality assessed with the NOS, with similar pooled estimates for studies scoring 8 (RR = 1.21, 95% CI 1.11–1.32; *P* < 0.0001; *I*^2^ ═ 93%) and 9 (RR = 1.23, 95% CI 1.09–1.40; *P* ═ 0.0008; *I*^2^ ═ 27%), and no evidence of subgroup differences (*P* for subgroup differences = 0.82). Abbreviations: RR: Risk ratio; CI: Confidence interval; CKD: Chronic kidney disease; eGFR: Estimated glomerular filtration rate; NOS: Newcastle–Ottawa Scale.

Subgroup analyses largely confirmed the initial findings. No significant differences were detected regarding the association between prediabetes and CKD risk across various definitions of prediabetes, including IFG, IGT, mildly elevated HbA1c, and their combinations (*P* for subgroup difference = 0.34; Figure 2B). Notably, a significant association was observed specifically for studies defining prediabetes by mildly elevated HbA1c (RR: 1.17, 95% CI: 1.03–1.33; *P* ═ 0.02; *I*^2^ ═ 88%; Figure 2B). Consistent results emerged for both prospective and retrospective studies (RR 1.16 vs. 1.28, *P* for subgroup difference = 0.25; Figure 3A), and across studies with mean ages < 57 and ≥ 57 years (RR 1.35 vs. 1.15, *P* for subgroup difference = 0.11; Figure 3B). Similarly, studies with a male proportion < 47% and ≥ 47% yielded comparable results (RR 1.22 vs. 1.19, *P* for subgroup difference = 0.82; Figure 4A), as did studies with mean follow-up durations < 6 years and ≥ 6 years (RR 1.38 vs. 1.14, *P* for subgroup difference = 0.16; Figure 4B). The association between prediabetes and CKD risk appeared stronger in studies where CKD was defined as eGFR < 60 mL/min/1.73 m^2^ and/or proteinuria compared to those where CKD was defined solely by eGFR < 60 mL/min/1.73 m^2^ (RR: 1.61 vs. 1.15), although this difference was not statistically significant (*P* for subgroup difference = 0.06; Figure 5A). Similar outcomes were noted for studies with NOS scores of 8 and 9 (RR 1.21 vs. 1.23, *P* for subgroup difference = 0.82; Figure 5B).

### Meta-regression analysis

Table 3 presents the univariate meta-regression results. The analysis indicated that mean age was inversely associated with the strength of the association between prediabetes and CKD risk (coefficient ═ –0.030, *P* = 0.004), accounting for a significant portion of the between-study heterogeneity (adjusted R^2^ ═ 67%). None of the other examined factors, including the proportion of men, mean BMI at baseline, follow-up length, or NOS score, significantly influenced the association between prediabetes and CKD risk (all *P* > 0.05).

**Table 1:** Characteristics of the included studies

**Study**	**Design**	**Country**	**Participant characteristics**	**Sample size**	**Mean age (years)**	**Men (%)**	**Mean BMI (kg/m^2^)**	**Criteria for the diagnosis of PreD**	**No. of participants with PreD**	**Follow-up duration (years)**	**Definition of CKD**	**No. of subjects with CKD**	**Variables adjusted**
Fox 2005	PC	USA	Community-based general population	2398	54.0	47.0	27.5	IFG or IGT	704	7.0	eGFR < 60 mL/min/1.73 m^2^	167	Age, sex, baseline eGFR, SBP, hypertension treatment, smoking, BMI, TC, HDL-C, and prevalent MI or CHF
Schöttker 2013	PC	Germany	Population-based sample of general adults (50--74 years)	3082	61.0	44.4	26.5	IFG or mildly elevated HbA1c	1054	8.0	eGFR < 60 mL/min/1.73 m^2^	678	Age, sex, baseline eGFR, BMI, SBP, TC, use of anti-hypertensive drugs, use of statins, smoking status, and history of self-reported CVD
Melsom 2015	PC	Norway	General population aged 50--62 years	1261	57.9	50.0	27.0	IFG or mildly elevated HbA1c	595	5.6	eGFR < 60 mL/min/1.73 m^2^	33	Age, sex, baseline eGFR, baseline use of ACEI/ARB, BMI, daytime systolic ambulatory BP, smoking, fasting insulin, physical exercise, and change in use of antihypertensive medication and change in FPG from baseline to follow-up
Tatsumi 2016	PC	Japan	Individuals aged 30--79 years undergoing a comprehensive medical check-up	2849	58.8	54.6	22.9	IFG or IGT	691	4.9	eGFR < 60 mL/min/1.73 m^2^	335	Age, sex, BMI, hypertension, dyslipidemia, smoking status, change in BMI and newly developed hypertension, dyslipidemia, CVD, cerebrovascular disease, and cancer during follow-up
Michishita 2017	RC	Japan	Middle-aged and older males receiving a periodic health check-up at a university healthcare center	303	52.2	100.0	23.4	IFG	29	6.0	eGFR < 60 mL/min/1.73 m^2^ and/or proteinuria	32	Age, BMI, baseline eGFR, smoking habits, and drinking habits
Jadhakhan 2018	RC	UK	Young adults (18--40 years) from the general population	40092	33.8	46.5	27.5	IFG or IGT	10561	1.7	eGFR < 60 mL/min/1.73 m^2^ and/or proteinuria	308	Age, sex, ethnic group, deprivation quintile, BMI categories, CVD, HF, AF, hypertension, and steroid use
Koshi 2018	RC	Japan	General population undergoing health check-ups	25109	48.0	57.8	22.5	IFG or mildly elevated HbA1c	10367	5.3	eGFR < 60 mL/min/1.73 m^2^ and/or proteinuria	2483	Age, sex, insulin sensitivity (SPISE), SBP, eGFR, and serum ALA level
Kim 2019	PC	South Korea	Adults from the general population	7728	52.0	52.6	NR	IFG, IGT, or mildly elevated HbA1c	2886	8.7	eGFR < 60 mL/min/1.73 m^2^	871	Age, sex, hypertension, obesity, regular physical activity, baseline eGFR, and MetS
Chen 2020	PC	China	Community-dwelling adults aged ≥ 40 years	7015	57.3	32.9	25.5	IFG, IGT, or mildly elevated HbA1c	4321	3.0	eGFR < 60 mL/min/1.73 m^2^	121	Age, sex, BMI, TC, TG, HDL-C, LDL-C, SBP, and DBP
Furukawa 2021	RC	Japan	Adults (≥20 years) from the general Japanese population who underwent a comprehensive health check-up	405487	50.0	61.9	22.8	IFG	116915	2.0	eGFR < 60 mL/min/1.73 m^2^ and/or proteinuria	25416	Age, sex, BMI, eGFR, hypertension, dyslipidemia, smoking, past history of CVD and stroke
Honigberg 2021	PC	UK	Adults aged 40--69 years from the general population	336709	56.3	44.6	27.1	Mildly elevated HbA1c	46911	11.1	ICD codes	8522	Age, sex, race, Townsend deprivation index, smoking, alcohol consumption, vegetable/fresh fruit intake, history of cancer, SBP, antihypertensive medication use, non-HDL cholesterol, cholesterol-lowering medication use, BMI, CRP, and UACR at baseline
Manouchehri 2022	PC	Spain	Adults (30--74 years) from primary care centers across Spain	1844	58.1	48.5	28.9	IFG or mildly elevated HbA1c	1072	5.0	eGFR < 60 mL/min/1.73 m^2^	149	Age, sex, smoking status, regular physical activity, alcohol consumption, adherence to Mediterranean diet score, daily fruit/vegetable consumption, WC, BMI, hypertension, TC, HDL-C, TG, and use of ACEIs or ARBs
Zhang 2023	RC	China	Adults (≥20 years) from the territory-wide Diabetes Surveillance Database	2003361	58.0	43.7	NR	IFG, IGT, or mildly elevated HbA1c	528948	7.8	eGFR < 60 mL/min/1.73 m^2^	18278	Age, sex, calendar year at baseline, LDL-C, TG/HDL-C ratio, Hb, albumin, use of lipid-regulating drugs and blood pressure-lowering drugs
Okawa 2023	RC	Japan	Non-diabetic Japanese citizens (aged 35 years or older) of Zentsuji city who participated in annual health checkups	7176	NR	40.4	NR	Mildly elevated HbA1c	3548	6.1	eGFR < 60 mL/min/1.73 m^2^	2374	Age, sex, BMI, self-reported drinking status, self-reported smoking status, hypertension, dyslipidemia, and residential area
Rooney 2025	PC	USA	Community-based adults aged 46–70 years	10310	57.0	44.0	27.2	IFG or mildly elevated HbA1c	6168	15.0	eGFR < 60 mL/min/1.73 m^2^	3288	Age, sex, race-center, smoking status, alcohol consumption, physical activity level, BMI, TC, HDL-C, lipid-lowering medication use, and hypertension

Abbreviations: NR: Not reported; PC: Prospective cohort; RC: Retrospective cohort; PreD: Prediabetes; IFG: Impaired fasting glucose; IGT: Impaired glucose tolerance; HbA1c: Glycated hemoglobin A1c; CKD: Chronic kidney disease; eGFR: Estimated glomerular filtration rate; BMI: Body mass index; SBP: Systolic blood pressure; DBP: Diastolic blood pressure; TC: Total cholesterol; HDL-C: High-density lipoprotein cholesterol; LDL-C: Low-density lipoprotein cholesterol; TG: Triglycerides; WC: Waist circumference; CRP: C-reactive protein; UACR: Urinary albumin-to-creatinine ratio; CVD: Cardiovascular disease; CHF: Congestive heart failure; MI: Myocardial infarction; HF: Heart failure; AF: Atrial fibrillation; ACEI: Angiotensin-converting enzyme inhibitor; ARB: Angiotensin II receptor blocker; SPISE: Single-point insulin sensitivity estimator; ALA: α-Linolenic acid; MetS: Metabolic syndrome; ICD: International Classification of Diseases.

**Table 2:** Evaluation of study quality using the Newcastle-Ottawa Scale

**Study**	**Representativeness of the exposed cohort**	**Selection of the non-exposed cohort**	**Ascertainment of exposure**	**Outcome not present at baseline**	**Control for age and sex**	**Control for other confounding factors**	**Assessment of outcome**	**Sufficient follow-up duration**	**Adequacy of follow-up of cohort**	**Total**
Fox 2005	1	1	1	1	1	1	1	1	1	9
Schöttker 2013	1	1	1	1	1	1	1	1	0	8
Melsom 2015	1	1	1	1	1	1	1	1	1	9
Tatsumi 2016	1	1	1	1	1	1	1	0	1	8
Michishita 2017	0	1	1	1	1	1	1	1	1	8
Jadhakhan 2018	1	1	1	1	1	1	1	0	1	8
Koshi 2018	0	1	1	1	1	1	1	1	1	8
Kim 2019	1	1	1	1	1	1	1	1	1	9
Chen 2020	1	1	1	1	1	1	1	0	1	8
Furukawa 2021	1	1	1	1	1	1	1	0	1	8
Honigberg 2021	1	1	1	1	1	1	0	1	1	8
Manouchehri 2022	1	1	1	1	1	1	1	1	1	9
Zhang 2023	0	1	1	1	1	1	1	1	1	8
Okawa 2023	0	1	1	1	1	1	1	1	1	8
Rooney 2025	1	1	1	1	1	1	1	1	1	9

### Publication bias

**Figure 6.**
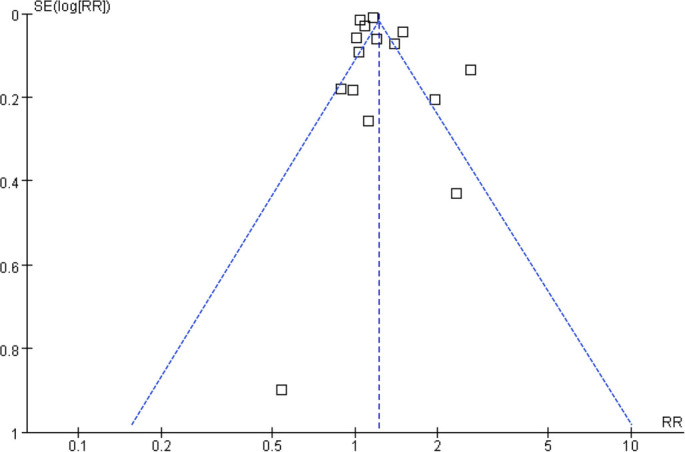
**Funnel plot assessing potential publication bias in the meta-analysis of the association between prediabetes (vs. normoglycemia) and CKD risk.** Each point represents an individual study (log risk ratio plotted against its standard error). The plot appears largely symmetrical around the pooled effect estimate (vertical dashed line), indicating little evidence of small-study effects or publication bias; this was supported by Egger’s regression test (*P* ═ 0.35).

As illustrated in Figure 6, funnel plots assessing the association between prediabetes and CKD risk in the general population were largely symmetrical, suggesting minimal publication bias. Egger’s test corroborated this observation, revealing no statistically significant bias (*P* ═ 0.35).

**Table 3:** Results of univariate meta-regression analysis

**Variables**	**RR for the association between prediabetes and the risk of CKD**			
	**Coefficient**	**95% CI**	***P* values**	**Adjusted R^2^**
Mean age (years)	--0.030	--0.048 to --0.012	0.004	67%
Men (%)	0.0038	--0.0126 to 0.0201	0.63	0%
Mean BMI (kg/m^2^)	0.0047	--0.1067 to 0.1162	0.93	0%
Follow-up duration (years)	--0.026	--0.072 to 0.021	0.26	0%
NOS	--0.098	--0.495 to 0.298	0.60	0%

Abbreviations: RR: Risk ratio; CKD: Chronic kidney disease; CI: Confidence interval; BMI: Body mass index; NOS: Newcastle-Ottawa Scale.

## Discussion

This meta-analysis synthesizes observational evidence indicating an association between prediabetes and a modestly increased risk of incident CKD in the general adult population. Analyzing data from over 2.8 million participants across 15 longitudinal cohorts, individuals with prediabetes exhibited approximately a 20% higher risk of incident CKD compared to those with normoglycemia. Although subgroup and meta-regression analyses did not uncover statistically significant effect modification by definitions of prediabetes, study design, follow-up duration, or methodological quality, substantial residual heterogeneity persisted across studies. The meta-regression analysis identified a significant inverse association between mean study age and the strength of the relationship between prediabetes and CKD risk, suggesting that cohorts with younger average ages tended to exhibit stronger associations. However, the age-stratified subgroup analysis (<57 vs. ≥ 57 years) did not reveal a statistically significant between-group difference, likely reflecting methodological discrepancies between the two approaches. Meta-regression treats age as a continuous variable, making it more sensitive to detecting linear trends across cohorts, whereas subgroup analysis relies on dichotomization using a median-based cutoff, which reduces statistical power and may obscure gradual age-related gradients. Collectively, these findings underscore that even mild glycemic dysregulation, below the diagnostic threshold for diabetes, may have clinically relevant renal consequences, highlighting the importance of early recognition and prevention.

Several biological mechanisms may elucidate the observed link between prediabetes and CKD development. Prediabetes is characterized by insulin resistance and low-grade hyperglycemia, both of which can induce glomerular and tubular injury through multiple metabolic and hemodynamic pathways [32, 33]. Chronic mild hyperglycemia increases oxidative stress and activates inflammatory cytokines, such as interleukin-6 and tumor necrosis factor-α, leading to endothelial dysfunction and microvascular damage [34, 35]. Additionally, insulin resistance and early dysglycemia may induce hemodynamic changes within the glomeruli, characterized by afferent arteriolar dilation and glomerular hyperfiltration, which represent early functional alterations that may precede structural injury and subsequent declines in glomerular filtration rate (GFR) [36]. Although hyperfiltration can be partially corrected through renin–angiotensin system blockade or sodium–glucose cotransporter 2 (SGLT2) inhibition, this meta-analysis focused on incident CKD defined by reduced eGFR and/or albuminuria and does not directly address treatment effects or longitudinal GFR dynamics [36]. Additional mechanisms include advanced glycation end-product accumulation [37], activation of the renin–angiotensin–aldosterone system [38], and disturbances in lipid metabolism [39], all of which can promote structural and functional decline of the kidneys. These processes create a pro-inflammatory and pro-fibrotic environment that predisposes the kidneys to early injury, even prior to the onset of overt diabetes [8]. The findings of this meta-analysis support the notion that CKD and diabetic kidney disease exist on a continuum that begins with prediabetic metabolic alterations. Importantly, emerging evidence suggests that the CKD risk associated with prediabetes may not be fully mediated by progression to overt diabetes. Large cohorts [20, 21] have demonstrated an increased incidence of CKD among individuals with prediabetes, even in the absence of diabetes progression. This observation implies that renal impairment arising in prediabetes may, at least in part, reflect pathophysiological pathways distinct from classical diabetic nephropathy, potentially involving early microvascular dysfunction, low-grade inflammation, or metabolic stress independent of sustained hyperglycemia.

Taken together, these analyses suggest a generally consistent direction of association; however, the PI highlights that the strength of the relationship between prediabetes and CKD risk is heterogeneous and may not be uniformly applicable across all populations. Subgroup analyses indicate a broadly consistent direction of association across various definitions of prediabetes, though the magnitude and statistical significance of the effects varied. Notably, several high-quality cohorts—particularly those defining prediabetes exclusively by mildly elevated HbA1c—reported weaker or null associations with CKD, contributing to substantial heterogeneity among studies. This variability may reflect differences in glycemic exposure as captured by fasting, post-load, and HbA1c-based definitions, as well as variations in baseline kidney function, follow-up duration, and residual confounding across cohorts. Consequently, the pooled estimate should be interpreted as an average association rather than a uniformly applicable risk, and clinical implications should be approached with caution. The absence of significant subgroup differences by study design or population characteristics further supports the generalizability of the findings across diverse demographic and geographic contexts. The inverse relationship between mean age and the effect size observed in the meta-regression may suggest a survivor or competing risk phenomenon, whereby older individuals accumulate multiple comorbidities that dilute the relative contribution of mild hyperglycemia to kidney risk [40]. Conversely, younger adults with prediabetes may experience a longer duration of exposure to dysglycemia, thereby amplifying its long-term impact on renal structure and function. However, these findings should be interpreted cautiously, as both the meta-regression and subgroup analyses are based on study-level mean age rather than individual participant data. The observed age-related pattern reflects differences between studies and should be considered exploratory. Age-specific pooled RRs from subgroup analyses are provided to illustrate this potential gradient, but they do not imply a definitive age threshold or causal modification effect.

The current analysis possesses several methodological strengths that enhance its reliability. First, the literature search was comprehensive and current, encompassing studies from multiple continents and incorporating recently published large-scale population-based cohorts. Second, all included studies employed longitudinal designs, allowing for the assessment of temporal relationships between prediabetes and subsequent CKD development, thereby minimizing the risk of reverse causality. Third, all analyses were adjusted for key confounders, including age, sex, BMI, blood pressure, and baseline kidney function, with pooled estimates derived exclusively from multivariable models. These strengths collectively provide robust support for the validity of the observed association. Nonetheless, several limitations should be acknowledged when interpreting the findings. First, despite the predominance of prospective cohorts, some included studies employed retrospective designs, which may introduce recall or selection bias. Second, substantial heterogeneity was observed across studies, likely reflecting differences in diagnostic criteria for prediabetes and CKD, population characteristics, follow-up duration, and residual confounding. Although meta-regression analyses identified mean age as a potential contributor, other sources of heterogeneity could not be fully explored due to limited reporting. Additionally, CKD definitions across studies were based on eGFR thresholds using different creatinine- or cystatin C–based equations, which may not represent identical levels of true measured GFR across regions or age groups. This variation could contribute to outcome misclassification and residual heterogeneity, particularly in younger and older populations. Furthermore, in older individuals, age-related declines in muscle mass and greater variability in body composition may reduce the accuracy of creatinine-based eGFR estimates [41], potentially leading to outcome misclassification and attenuated associations in cohorts with a higher mean age. Third, individual participant data were not available, precluding harmonized reclassification of prediabetes subtypes, stratification by ethnicity, or adjustment for medication use, lifestyle factors, or comorbidities. Fourth, although most studies adjusted for major confounders, residual confounding by unmeasured variables, such as dietary habits, socioeconomic status, or family history, cannot be excluded [42]. Fifth, the nature of the analysis is observational and cannot establish a causal relationship between prediabetes and CKD. It remains possible that prediabetes serves as a marker of broader metabolic dysfunction rather than a direct cause of renal decline. Moreover, while the pooled RR was statistically significant, its clinical impact is modest, and translation into absolute risk differences was not attempted due to substantial heterogeneity in baseline CKD risk across populations. Lastly, although funnel plots and Egger’s test did not suggest significant publication bias, these methods have limited power in the presence of substantial heterogeneity and a modest number of studies; therefore, small-study effects and selective reporting cannot be excluded, and these assessments should be regarded as exploratory.

The clinical implications of these findings are significant. Prediabetes is highly prevalent worldwide, affecting approximately one-third of adults, and is increasingly recognized as a stage at which vascular and microvascular complications may begin [43]. The observed 20% increased risk of CKD underscores the need for clinicians to regard prediabetes not only as a precursor to diabetes but also as a condition with independent renal implications. Early identification of individuals with prediabetes provides an opportunity for lifestyle modifications, weight management, blood pressure control, and optimization of lipid and glycemic profiles—all measures known to mitigate microvascular injury [44]. While interventional evidence is lacking, the observed association may be considered hypothesis-generating and consistent with existing guideline-based risk assessment practices rather than implying new monitoring recommendations. At the population level, these results reinforce the importance of integrating kidney health into broader chronic disease prevention frameworks targeting metabolic risk. Future research should focus on elucidating the causal pathways linking prediabetes to renal injury using individual-level pooled data and longitudinal trajectory analyses. Standardized diagnostic criteria for both prediabetes and CKD would improve comparability across studies, while mechanistic studies could clarify the relative contributions of hyperglycemia, insulin resistance, and other metabolic abnormalities to kidney dysfunction. Intervention trials assessing whether intensive lifestyle modification or pharmacologic therapy in prediabetic individuals can prevent CKD onset are also warranted. Such evidence would help determine whether early management of dysglycemia confers renal protection beyond its established cardiovascular benefits. These findings from the meta-analysis suggest that early stages of glycemic dysregulation may be associated with increased renal risk. However, interventional evidence for CKD prevention in prediabetes remains limited, and clinical decisions should consider the modest magnitude of risk, individual patient context, and existing guideline recommendations.

## Conclusion

In conclusion, this meta-analysis reveals that prediabetes is linked to a modestly elevated risk of CKD within the general population, with a potentially stronger correlation observed among younger cohorts. It is important to note that these findings indicate an association rather than causation and should be interpreted with caution due to the observational design and considerable heterogeneity. Overall, the results suggest a potential connection between early dysglycemia and increased kidney risk, highlighting the need for further exploration in well-designed prospective studies and randomized interventional trials.

## Supplemental data

**Detailed search strategy for each database**

**PubMed**

#1 “Prediabetic State”[Mesh] OR prediabetes[tiab] OR “pre-diabetes”[tiab] OR prediabetic[tiab] OR “pre-diabetic”[tiab] OR “borderline diabetes”[tiab] OR “impaired fasting glucose”[tiab]OR “impaired glucose tolerance”[tiab] OR IFG[tiab] OR IGT[tiab]

#2 “Kidney Diseases”[Mesh] OR “Renal Insufficiency, Chronic”[Mesh] OR “Glomerular Filtration Rate”[Mesh] OR “chronic kidney disease”[tiab] OR CKD[tiab] OR “renal function”[tiab] OR “chronic renal failure”[tiab]

#3 “Cohort Studies”[Mesh] OR cohort[tiab] OR prospective[tiab] OR retrospective[tiab] OR prospectively[tiab] OR retrospectively[tiab] OR follow[tiab] OR followed[tiab] OR “follow-up”[tiab] OR longitudinal[tiab] OR risk[tiab] OR incidence[tiab]

#4 #1 AND #2 AND #3

Filters: Humans, Publication date: database inception – 2025/09/28

**Embase**

#1 ‘prediabetes’/exp OR prediabetes:ab,ti OR ‘pre-diabetes’:ab,ti OR prediabetic:ab,ti OR ‘pre-diabetic’:ab,ti OR ‘borderline diabetes’:ab,ti OR ‘impaired fasting glucose’:ab,ti OR ‘impaired glucose tolerance’:ab,ti OR IFG:ab,ti OR IGT:ab,ti

#2 ‘chronic kidney disease’/exp OR ‘chronic renal failure’/exp OR ‘renal function’/exp OR ‘glomerular filtration rate’/exp OR ‘chronic kidney disease’:ab,ti OR CKD:ab,ti OR ‘renal function’:ab,ti OR ‘chronic renal failure’:ab,ti

#3 ‘cohort analysis’/exp OR ‘prospective study’/exp OR ‘retrospective study’/exp OR cohort:ab,ti OR prospective:ab,ti OR retrospective:ab,ti OR prospectively:ab,ti OR retrospectively:ab,ti OR follow:ab,ti OR followed:ab,ti OR ‘follow up’:ab,ti OR longitudinal:ab,ti OR risk:ab,ti OR incidence:ab,ti

#4 #1 AND #2 AND #3

Limits: Humans, publication year ≤ 2025

**Web of Science**

TS=((“prediabetes” OR “pre-diabetes” OR “prediabetic” OR “pre-diabetic” OR “prediabetic state” OR “borderline diabetes” OR “impaired fasting glucose” OR “impaired glucose tolerance” OR “IFG” OR “IGT”)

AND

(“chronic kidney disease” OR “CKD” OR “glomerular filtration rate” OR “renal function” OR “chronic renal failure”)

AND

(“cohort” OR “prospective” OR “retrospective” OR “prospectively” OR “retrospectively” OR “follow” OR “followed” OR “follow-up” OR “longitudinal” OR “risk” OR “incidence”))

Refine by: Document Type = Article; Species = Humans; Timespan = All years to 2025-09-28.

## Data Availability

All data generated or analyzed during this study are included in this published article.

## References

[1] Francis A, Harhay MN, Ong ACM, Tummalapalli SL, Ortiz A, Fogo AB (2024). Chronic kidney disease and the global public health agenda: an international consensus. Nat Rev Nephrol.

[2] Shao Y, Fan Y, Gao J, Meng H, Wang M, Shi Q (2025). Prevalence, awareness, and treatment of chronic kidney disease among adults in Yunnan Province, China: findings from the 2023 chronic disease and risk factors surveillance. Front Public Health.

[3] Zoccali C, Mallamaci F, Adamczak M, de Oliveira RB, Massy ZA, Sarafidis P (2023). Cardiovascular complications in chronic kidney disease: a review from the European Renal and Cardiovascular Medicine Working Group of the European Renal Association. Cardiovasc Res.

[4] Levey AS, de Jong PE, Coresh J, El Nahas M, Astor BC, Matsushita K (2011). The definition, classification, and prognosis of chronic kidney disease: a KDIGO Controversies Conference report. Kidney Int.

[5] Gembillo G, Ingrasciotta Y, Crisafulli S, Luxi N, Siligato R, Santoro D (2021). Kidney Disease in Diabetic Patients: From Pathophysiology to Pharmacological Aspects with a Focus on Therapeutic Inertia. Int J Mol Sci.

[6] Echouffo-Tcheugui JB, Perreault L, Ji L, Dagogo-Jack S (2023). Diagnosis and Management of Prediabetes: a Review. JAMA.

[7] Davidson MB (2022). Historical review of the diagnosis of prediabetes/intermediate hyperglycemia: Case for the international criteria. Diabetes Res Clin Pract.

[8] Rico Fontalvo J, Soler MJ, Daza Arnedo R, Navarro-Blackaller G, Medina-González R, Rodríguez Yánez T (2024). Prediabetes and CKD: Does a causal relationship exist. Nefrologia (Engl Ed).

[9] Jadhakhan F, Marshall T, Gill P (2015). A systematic review investigating the cumulative incidence of chronic kidney disease in young adults with impaired glucose tolerance. Syst Rev.

[10] Echouffo-Tcheugui JB, Narayan KM, Weisman D, Golden SH, Jaar BG (2016). Association between prediabetes and risk of chronic kidney disease: a systematic review and meta-analysis. Diabet Med.

[11] Michishita R, Matsuda T, Kawakami S, Tanaka S, Kiyonaga A, Tanaka H (2017). Hypertension and hyperglycemia and the combination thereof enhances the incidence of chronic kidney disease (CKD) in middle-aged and older males. Clin Exp Hypertens.

[12] Jadhakhan F, Marshall T, Ryan R, Gill P (2018). Risk of chronic kidney disease in young adults with impaired glucose tolerance/impaired fasting glucose: a retrospective cohort study using electronic primary care records. BMC Nephrol.

[13] Koshi T, Sagesaka H, Sato Y, Hirabayashi K, Koike H, Yamauchi K (2018). Elevated haemoglobin A1c but not fasting plasma glucose conveys risk of chronic kidney disease in non-diabetic individuals. Diabetes Res Clin Pract.

[14] Kim GS, Oh HH, Kim SH, Kim BO, Byun YS (2019). Association between prediabetes (defined by HbA1(C), fasting plasma glucose, and impaired glucose tolerance) and the development of chronic kidney disease: a 9-year prospective cohort study. BMC Nephrol.

[15] Chen C, Liu G, Yu X, Yu Y (2020). Association between Prediabetes and Renal Dysfunction from a Community-based Prospective Study. Int J Med Sci.

[16] Furukawa M, Onoue T, Kato K, Wada T, Shinohara Y, Kinoshita F (2021). Prediabetes is associated with proteinuria development but not with glomerular filtration rate decline: a longitudinal observational study. Diabet Med.

[17] Honigberg MC, Zekavat SM, Pirruccello JP, Natarajan P, Vaduganathan M (2021). Cardiovascular and Kidney Outcomes Across the Glycemic Spectrum: Insights From the UK Biobank. J Am Coll Cardiol.

[18] Manouchehri M, Cea-Soriano L, Franch-Nadal J, Ruiz A, Goday A, Villanueva R (2022). Heterogeneity in the association between prediabetes categories and reduction on glomerular filtration rate in a 5-year follow-up. Sci Rep.

[19] Okawa Y, Suzuki E, Mitsuhashi T, Tsuda T, Yorifuji T (2023). A population-based longitudinal study on glycated hemoglobin levels and new-onset chronic kidney disease among non-diabetic Japanese adults. Sci Rep.

[20] Zhang X, Wu H, Fan B, Shi M, Lau ESH, Yang A (2023). The role of age on the risk relationship between prediabetes and major morbidities and mortality: Analysis of the Hong Kong diabetes surveillance database of 2 million Chinese adults. Lancet Reg Health West Pac.

[21] Rooney MR, Wallace AS, Echouffo Tcheugui JB, Fang M, Hu J, Lutsey PL (2025). Prediabetes is associated with elevated risk of clinical outcomes even without progression to diabetes. Diabetologia.

[22] Higgins J, Thomas J, Chandler J, Cumpston M, Li T, Page M (2021). Cochrane Handbook for Systematic Reviews of Interventions version 6.2. The Cochrane Collaboration.

[23] Wells GA, Shea B, O’Connell D, Peterson J, Welch V, Losos M (2010). The Newcastle–Ottawa Scale (NOS) for assessing the quality of nonrandomised studies in meta-analyses. http://www.ohri.ca/programs/clinical/_epidemiology/oxford.asp.

[24] Zhang J, Yu KF (1998). What’s the relative risk? A method of correcting the odds ratio in cohort studies of common outcomes. JAMA.

[25] Higgins JP, Thompson SG (2002). Quantifying heterogeneity in a meta-analysis. Stat Med.

[26] Marušić MF, Fidahić M, Cepeha CM, Farcaş LG, Tseke A, Puljak L (2020). Methodological tools and sensitivity analysis for assessing quality or risk of bias used in systematic reviews published in the high-impact anesthesiology journals. BMC Medical Research Methodology.

[27] Egger M, Davey Smith G, Schneider M, Minder C (1997). Bias in meta-analysis detected by a simple, graphical test. BMJ.

[28] Fox CS, Larson MG, Leip EP, Meigs JB, Wilson PW, Levy D (2005). Glycemic status and development of kidney disease: the Framingham Heart Study. Diabetes Care.

[29] Schöttker B, Brenner H, Koenig W, Müller H, Rothenbacher D (2013). Prognostic association of HbA1c and fasting plasma glucose with reduced kidney function in subjects with and without diabetes mellitus. Results from a population-based cohort study from Germany. Prev Med.

[30] Melsom T, Schei J, Stefansson VT, Solbu MD, Jenssen TG, Mathisen UD (2015). Prediabetes and Risk of Glomerular Hyperfiltration and Albuminuria in the General Nondiabetic Population: a Prospective Cohort Study. Am J Kidney Dis.

[31] Tatsumi Y, Morimoto A, Soyano F, Shimoda T, Miyamatsu N, Ohno Y (2016). Risk of proteinuria among individuals with persistent borderline diabetes: the Saku study. Diabetol Int.

[32] Ping WX, Hu S, Su JQ, Ouyang SY (2024). Metabolic disorders in prediabetes: From mechanisms to therapeutic management. World J Diabetes.

[33] Tuttle KR, Agarwal R, Alpers CE, Bakris GL, Brosius FC, Kolkhof P (2022). Molecular mechanisms and therapeutic targets for diabetic kidney disease. Kidney Int.

[34] Kim JA, Montagnani M, Koh KK, Quon MJ (2006). Reciprocal relationships between insulin resistance and endothelial dysfunction: molecular and pathophysiological mechanisms. Circulation.

[35] Giri B, Dey S, Das T, Sarkar M, Banerjee J, Dash SK (2018). Chronic hyperglycemia mediated physiological alteration and metabolic distortion leads to organ dysfunction, infection, cancer progression and other pathophysiological consequences: an update on glucose toxicity. Biomed Pharmacother.

[36] De Cosmo S, Menzaghi C, Prudente S, Trischitta V (2013). Role of insulin resistance in kidney dysfunction: insights into the mechanism and epidemiological evidence. Nephrol Dial Transplant.

[37] Rabbani N, Thornalley PJ (2018). Advanced glycation end products in the pathogenesis of chronic kidney disease. Kidney Int.

[38] Lovshin JA, Boulet G, Lytvyn Y, Lovblom LE, Bjornstad P, Farooqi MA (2018). Renin-angiotensin-aldosterone system activation in long-standing type 1 diabetes. JCI Insight.

[39] Wei S, Fu Y, Zeng Y, Wu W, Cai J, Dong Z (2025). Lipid metabolism in AKI and AKI-CKD transition: Dysregulation, lipotoxicity and therapeutic potential. Pharmacol Ther.

[40] Ravender R, Roumelioti ME, Schmidt DW, Unruh ML, Argyropoulos C (2024). Chronic Kidney Disease in the Older Adult Patient with Diabetes. J Clin Med.

[41] Gaillard F, Rabah MO, Aubert O, Garcelon N, Neuraz A, Legendre C (2025). Impact of Muscle Mass on the Performance of Creatinine-Based eGFR Equations and Mortality Risk Assessment After Kidney Transplantation. J Cachexia Sarcopenia Muscle.

[42] Crews DC, Kuczmarski MF, Miller ER 3rd, Zonderman AB, Evans MK, Powe NR (2015). Dietary habits, poverty, and chronic kidney disease in an urban population. J Ren Nutr.

[43] Baranowska-Jurkun A, Matuszewski W, Bandurska-Stankiewicz E (2020). Chronic Microvascular Complications in Prediabetic States-An Overview. J Clin Med.

[44] Tuso P (2014). Prediabetes and lifestyle modification: time to prevent a preventable disease. Perm J.

